# The effects of pressure- versus volume-controlled ventilation on ventilator work of breathing

**DOI:** 10.1186/s12938-020-00815-x

**Published:** 2020-09-15

**Authors:** Mojdeh Monjezi, Hamidreza Jamaati

**Affiliations:** Chronic Respiratory Diseases Research Center (CRDRC), National Research Institute of Tuberculosis and Lung Diseases (NRITLD), Shahid Beheshti University of Medical Sciences, Tehran, Iran

**Keywords:** Work of breathing, Inspiratory flow waveform, Volume-controlled ventilation, Pressure-controlled ventilation, Resistive pressure drop, Mechanical work

## Abstract

**Background:**

Measurement of work of breathing (WOB) during mechanical ventilation is essential to assess the status and progress of intensive care patients. Increasing ventilator WOB is known as a risk factor for ventilator-induced lung injury (VILI). In addition, the minimization of WOB is crucial to facilitate the weaning process. Several studies have assessed the effects of varying inspiratory flow waveforms on the patient’s WOB during assisted ventilation, but there are few studies on the different effect of inspiratory flow waveforms on ventilator WOB during controlled ventilation.

**Methods:**

In this paper, we analyze the ventilator WOB, termed mechanical work (MW) for three common inspiratory flow waveforms both in normal subjects and COPD patients. We use Rohrer’s equation for the resistance of the endotracheal tube (ETT) and lung airways. The resistance of pulmonary and chest wall tissue are also considered. Then, the resistive MW required to overcome each component of the respiratory resistance is computed for square and sinusoidal waveforms in volume-controlled ventilation (VCV), and decelerating waveform of flow in pressure-controlled ventilation (PCV).

**Results:**

The results indicate that under the constant I:E ratio, a square flow profile best minimizes the MW both in normal subjects and COPD patients. Furthermore, the large I:E ratio may be used to lower MW. The comparison of results shows that ETT and lung airways have the main contribution to resistive MW in normals and COPDs, respectively.

**Conclusion:**

These findings support that for lowering the MW especially in patients with obstructive lung diseases, flow with square waveforms in VCV, are more favorable than decelerating waveform of flow in PCV. Our analysis suggests the square profile is the best choice from the viewpoint of less MW.

## Background

Measurement of work of breathing (WOB) during mechanical ventilation is essential to assess the status and progress of intensive care patients. WOB can be performed by the ventilator, during controlled mechanical ventilation, by the patient’s respiratory muscles during spontaneous breathing; or by a combination of both, as in assisted mechanical ventilation. So, WOB includes both patient’s WOB and ventilator WOB [1]. Patient’s (WOB) has been proved to be a predictor of weaning [2–4]; however, since it requires esophageal pressure measuring, the procedure is rarely used in routine practice. In contrast, the ventilator WOB which is identical to the mechanical work done by the ventilator can be continuously calculated at the bedside. Ventilator WOB can be an informative parameter later on during assisted ventilation when effort is required for triggering and maintaining adequate inspiratory flows. The concept of mechanical power (MP) as a measure for the development of ventilator-induced lung injury (VILI) is a promising idea [5]. Moreover, recently it has been found that MP of the ventilator normalized to lung–thorax compliance was independently correlated with weaning outcome [6]. Therefore, careful adjustment of the ventilator settings is necessary to minimize MP. MP is defined as the energy delivered to the respiratory system per unit time [7]. Using the term of mechanical work (MW) instead of the energy delivered to the respiratory system by ventilator per breath (ventilator WOB), MP is MW multiplied by respiratory rate (RR) [7]. MW may be defined as the area between the inspiratory limb of pressure–volume curve and the volume axis [8]. Traditional strategies to overcome ventilator‐associated lung injury (VALI) include limiting the tidal volume to prevent overinflation and the use of PEEP to prevent cyclic alveolar collapse. Recently, MP is defined as a unified index for measuring the risk of VILI in patients under mechanical ventilation [7]. So, it is interesting to estimate which inspiratory flow waveform can minimize MP. The components of MW consist of work needed to overcome both dissipative and elastic resistance of the respiratory system. The elastic work does not dependent on the characteristics of the flow and can be found through a constant parameter for a given inspiratory volume. The flow-dependent component of the MW is work done by dissipative resistance resulting from the frictional stresses on the gas flow in the airways.

A decelerating flow pattern is suggested for patients with restrictive lung diseases such as acute respiratory distress syndrome (ARDS) and acute lung injury, because of reducing the risk of VILI and more even gas distribution [9, 10]. But its shortened expiratory time is not suggested for obstructive pulmonary diseases. For these patients with asthma or chronic obstructive pulmonary disease (COPD), a square waveform is suggested [11, 12].

Conflicting results have been obtained, from both animal models and clinical observations, as to the relative effectiveness of different inspiratory flow patterns during mechanical ventilation. The selection of decelerating flow has been claimed to favor better gas exchange and respiratory mechanics when compared with the constant square inspiratory flow [13–22]. In addition, pressure-controlled ventilation (PCV) has some advantages [18] over volume-controlled ventilation (VCV), for example, its resulting square wave pressure waveform provides the maximum inspiratory pressure for the entire inspiratory time favors lung recruitment. This beneficial effect of pressure-controlled ventilation may be useful to overcome atelectasis. However, tidal recruitment increases mechanical stress on the lungs, and this may promote VILI especially in preterm infants [23].

Dembinski et al. [24] compared the effect of decelerating, square, and a fixed combination of both flow waveforms on the distribution of ventilation and perfusion in an animal model of acute lung injury. They reported that contrary to the hypothesis, square waveform provides a more favorable distribution of ventilation and perfusion, and hence better oxygenation when compared with decelerating or combined flow waveforms in this model of ALI. Their different conclusion may have originated from choosing a lower tidal volume for the square rather than decelerating or combined flow waveforms.

Roth et al. [25] reported that decelerating inspiratory flow had no beneficial effects on pulmonary gas exchange when compared with the square inspiratory flow while an increase in mean airway pressure in pressure‐controlled ventilation may raise the potential risk of VILI. Some other experimental studies [26, 27] showed that there are no differences between square or decelerating flow waveform in oxygenation. Antonaglia et al. mathematical model [28] showed that both ventilatory modes provided similar gas distribution, but in square flow, peak pressures were higher in the sicker compartment with respect to decelerating flow. They demonstrated that less pressure variability in PCV could reduce the potential VILI.

A previous study showed that constant flow minimizes the work on the endotracheal tube [29]. Wong et al. found that mechanical ventilated patients with acute respiratory failure show improved respiratory mechanics with decelerating inspiratory waveform [17]. Moreover, the clinical tests of Yang et al. [16] on critically ill patients with COPD showed that ventilator WOB values were reduced with the decelerating waveform.

In these studies, the decelerating flow profile was generated by VCV instead of PCV. Kallet et al. [30] and Cinnella et al. [31] assessed the effects of PCV versus VCV on patient’s WOB during assisted ventilation and conclude that inspiratory assistance delivered at a constant pressure reduces the respiratory work rate more effectively than assist control ventilation. However, to the best of our knowledge, the effects of PCV versus VCV on ventilator WOB are not examined yet.

In this study, we seek to compare different inspiratory waveforms from the viewpoint of MW imposed. We calculate ventilator WOB, which remains a helpful parameter of the patient’s WOB later on during assisted ventilation when an effort is required for triggering and maintaining adequate inspiratory flows.

In the methods section, we explain how the mechanical work is computed and compared between square, sine, and decelerating waveforms of inspiratory flow. The resistive work then computed to find the most favorable flow pattern for ventilated patients with minimal MW.

## Results

We first evaluate how the MW of ventilation changes for different lung conditions corresponding to changes in the waveform. Table 1 presents the MW results for different inspiratory flow waveforms with two different rise times and three types of lung mechanics, namely normal, resistive, and obstructive. Assuming laminar fully developed flow, the MW in the reference condition (RR = 15 L/min, *I*:*E* = 1/4) is calculated through Eqs. 7, 10, and 11. The results reveal that decelerating flow waveforms have the largest MW in both normal and restrictive lung conditions, while for the lung with the obstructive condition, sinusoidal waveform results in maximum MW. On the other hand, the smallest MW in all lung conditions correspond to the square waveform.

**Table 1:** MW of ventilation (cmH2O.L) with different flow waveforms (decelerating, square, and sinusoidal) with three types of lung mechanics

Pressure setting	Inspiratory flow waveform	Normal lung R 10 cmH2O/L/s, C 0.1 L/cmH2O	Restrictive lung diseases R 10 cmH2O/L/s, C 0.05 L/cmH2O	Obstructive lung diseases R 20 cmH2O/L/s, C 0.1 L/cmH2O
*t*_r_ 0.01 s, PS 10 cmH2O	Decelerating Square Sinusoidal	6.17 5.81 6.71	4.18 3.57 3.99	3.86 3.77 4.48
*t*_r_ 0.1 s, PS 10 cmH2O	Decelerating Square Sinusoidal	4.98 4.40 5.08	3.11 2.44 2.72	3.23 2.99 3.55
*t*_r_ 0.01 s, PS 20 cmH2O	Decelerating Square Sinusoidal	24.69 23.23 26.85	16.71 14.28 15.95	15.44 15.09 17.91
*t*_r_ 0.1 s, PS 20 cmH2O	Decelerating Square Sinusoidal	19.92 17.57 20.31	12.44 9.74 10.88	12.90 11.96 14.20

Also illustrated 2 levels of pressure support and 2 rise times. The square flow waveform has the smallest MW for all lung conditions. In normal and restrictive lungs the largest MW results from decelerating flow, while in obstructive lung condition the sinusoidal flow has the largest MW

As shown in Table 1, both in normal or diseased lungs, square flow waveform has the smallest MW. This result is confirmed for both two levels of pressure support and rise times. Previous studies also suggest similarly that to lower MW for patients with obstructive lung diseases the square flow is preferred to decelerating flow [11, 12]. It is noteworthy that setting a faster rise time or higher pressure support increases the MW of ventilation due to an increase in tidal volume. These differences in tidal volumes also explain variations of the MW between normal and diseased lungs. Our aim here, in contrast, was to identify the sensitivity of MW to flow waveform when tidal volume is fixed. Then the impact of tidal volume on resistive MW is assessed.

As explained in the Methods section, the total resistive MW comprised the resistive MW on ETT, pulmonary and chest wall tissues, and lung airways. Table 2 demonstrates the total resistive MW and its components for three levels of 0.5, 1, and 2 L of tidal volumes and the reference condition for a normal lung with a constant *C* = 0.1 L/cmH2O, and pressure rising time, *t*_r_ = 0.01 s. It should be noted that respiratory resistance is flow-dependent.

**Table 2:** Resistive MW of ventilation (cmH2O.L) assuming different flow waveforms, i.e., decelerating, square and sinusoidal, with three levels of tidal volumes

Inspiratory flow waveform	*V*_T_ = 0.5 L	*V*_T_ = 1 L	*V*_T_ = 2 L
Decelerating	Total 2.81 ETT 1.32 Tissues 0.89 Airways 0.60	Total 14.55 ETT 8.60 Tissues 3.43 Airways 2.52	Total 84.72 ETT 59.88 Tissues 13.27 Airways 11.57
Square	Total 2.31 ETT 1.01 Tissues 0.78 Airways 0.52	Total 12.64 ETT 7.20 Tissues 3.16 Airways 2.28	Total 77.70 ETT 54.21 Tissues 12.65 Airways 10.84
Sinusoidal	Total 3.20 ETT 1.57 Tissues 0.97 Airways 0.66	Total 18.39 ETT 11.50 Tissues 3.90 Airways 2.99	Total 118.18 ETT 87.77 Tissues 15.62 Airways 14.79

For the volumes below 1 L, the smallest resistive work is obtained by square flow, while the sinusoidal flow has the smallest resistive work for volumes equal or higher than 1 L

According to Table 2, the resistive work on ETT is the major component of total resistive work and its contribution increased by increasing the inspiratory flow rate. Similar to the previous results in Table 1 (normal lung column), sinusoidal and square flow waveform has the largest and smallest resistive work in all ranges of volumes, respectively.

**Fig. 1:**
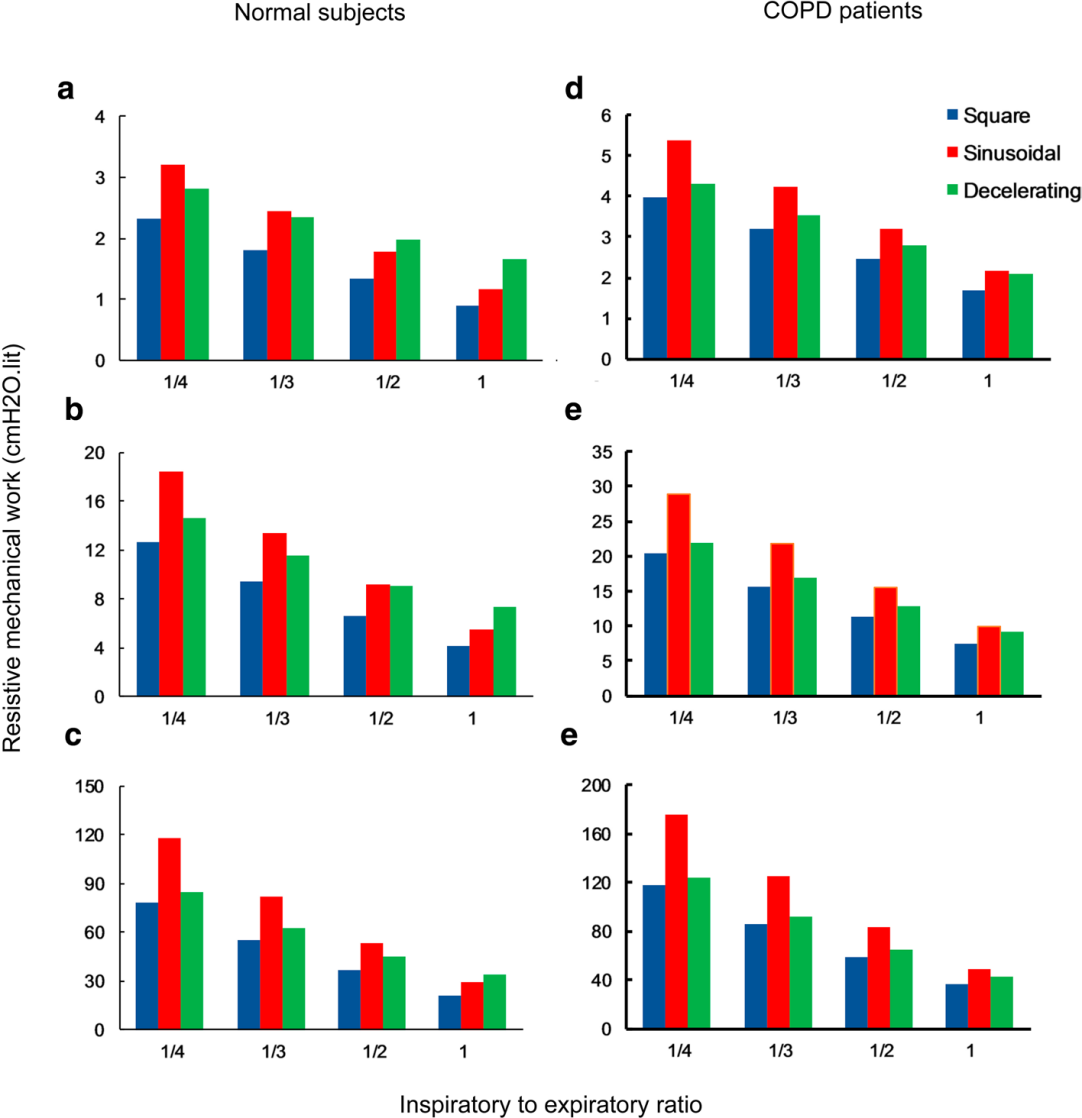
Resistive MW of the ventilator (cmH2O.L) assuming different flow waveforms (square, sinusoidal, and decelerating) for both normal subjects (left column) and COPD patients (right column) with RR = 15/min and different I:E ratios under different tidal volumes; a, d) *V*_T_ = 0.5 L, b, e) *V*_T_ = 1 L, c, f) *V*_T_ = 2 L; increasing I:E ratio in the same RR would decrease MW for all flow profiles. For a fixed I:E ratio, the square profile has the smallest MW for all *V*_T_ = 0.5 L (see blue bars) while the largest MW is achieved by sinusoidal or decelerating depending on I:E ratio. Resistive MW in COPD patients is about two times higher than that in normals

The foregoing analysis was under the respiratory rate of 15/min and *I*:*E* of 1/4 which corresponds to *t*_*i*_ of 1 s. We have considered the same inspiration time in all comparisons. Our next question is to examine how MW changes with different inspiration times. To answer this question, we compare also the results for *I*:*E* ratios of 1/3, 1/2, and 1/1 and a constant respiratory rate of 15/min (i.e., t_i_ of 1.25, 1.66, and 2.5 s). For multiple rates of tidal volume, the resistive MW computations as a function of I:E ratio for different flow waveforms are shown in Fig. 1 for both normal subjects and COPD patients.

We observe firstly in both (Fig. 1) that lower tidal volumes have lower MW. Moreover, we infer that increasing I:E ratio in a constant tidal volume will decrease resistive MW for each flow profile. These results suggest that to lower MW, a square flow needs to be selected. On the other hand, if a decelerating profile is to be selected, by increasing the I:E ratio from 1:4 to 1:1 the MW can be reduced. The largest MW is achieved either by sinusoidal and decelerating flow waveform depending on I:E ratio, sinusoidal under I:E = 1:4 or 1:3 and decelerating under I:E = 1:2 or 1:1. Similar results can be observed both for normal subjects and COPD patients except that the resistive MW in COPD patients is about two times higher than that in normal subjects.

**Fig. 2:**
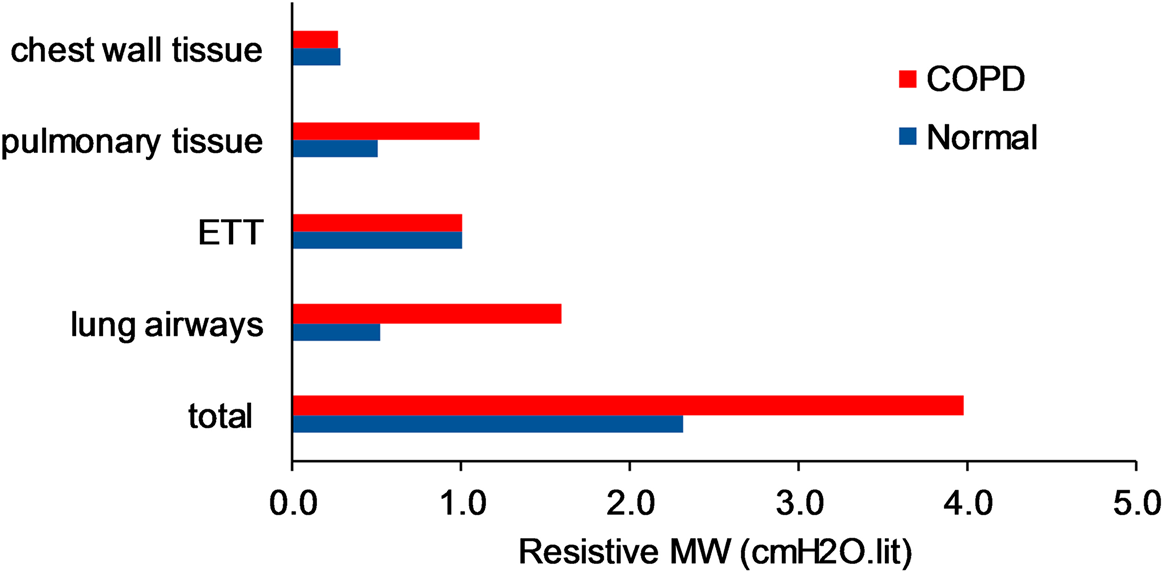
Partitioning of resistive MW of the ventilator (cmH2O.L) applied due to different components of the respiratory system assuming a square profile with *V*_T_ = 0.5 L and the reference condition for both normal subjects and COPDs. The work exerted to the ETT and lung airways is the major components of the resistive MW in normals and COPDs, respectively

The resistive MW due to the different resistive compartments of the respiratory system resistance, i.e., lung airways, ETT, pulmonary tissue, and chest wall tissue is shown in Fig. 2 for a square profile with a tidal volume of 0.5 L and the reference condition. As shown the work exerted on the ETT and lung airways is the major components of the resistive MW in normals and COPDs, respectively. Moreover, the chest wall tissue has the same contribution in resistive MW in normals and COPDs while the pulmonary tissue contribution in COPDs is two times more than normals.

Above results suggest that the optimum flow waveform to minimize MW of the ventilator is the square one with I:E = 1:1. Moreover, the resistive MW applied to the lung can be compared to make a decision about the best flow waveform which minimizes the risk of VILI. We also compared the resistive MW on the lung without considering the ETT resistance and found that it has a similar trend to the total resistive MW. It suggests again that to lower resistive work on the lung, a square profile better minimizes the MW for all I:E ratios compared to sinusoidal and decelerating profiles.

## Discussion

The resistive MW of ventilation is dependent on the flow waveform. Since a linear relationship between flow rate and pressure loss is not valid for large Reynolds numbers, Re > 2300, Rohrer’s equation is used for the resistance of lung airways and also ETT. Our results reveal that for all tidal volumes, the square profile has the smallest MW.

According to our findings, a square profile is recommended to lower resistive MW on the mechanically ventilated patients either normal or COPD. On the other hand, if a decelerating profile is selected for a reason, you can increase the I:E ratio as much as possible MW is lowered. It should be noted that these results pertain to a respiratory rate of 15 per minute although changes in the respiratory rate do not impact our findings.

In several clinical trials, the WOB of the decelerating waveform is reported to be lower than that of square or sinusoidal inspiratory waveform of flow [15, 16]. One reason for these conflicting results may be that the decelerating flow waveform in both of these studies is a linear profile produced during VCV, not an exponential profile produced during PCV. However, we repeated our computations for a linear decelerating profile (VCV) and found that its resistive MW is still higher than the square one. So, other reasons should have existed. For example, Al-saady and Bennett [15] calculated WOB by the product of tidal volume and peak pressure generated while we have integrated the product of flow and pressure over time mathematically. Their result shows that with tidal volume being kept constant, the decelerating waveform produced statistically significant reduction of peak pressure and consequently the product of tidal volume and peak pressure. Moreover, Wong et al. also show improved respiratory mechanics, but no statistically significant difference in WOB [17]. One reason for their different result may be that they adjusted the initial peak flow to attain a constant mean airway pressure for the three different waveforms instead of using a constant tidal volume. However, our result is in agreement with Polese et al. [29], in which the work on the endotracheal tube is reported to be minimum with a constant square profile. Besides, PCV is reported to be more effective than VCV in the reduction of patient’s WOB [30, 31], which is in contrast to our result about ventilator WOB. This disagreement is chiefly because the patients in these clinical tests were on assisted ventilation in which patient–ventilator synchrony is an important determinant of reducing WOB. It is also reported that [32] patient-triggered breaths during PCV may increase tidal volume unless the inspiratory pressure is reduced, which in turn may decrease the peak inspiratory flow rate. Therefore, PCV offers no general advantage in reducing WOB, compared to VCV, and in some patients does not allow for the control of tidal volume to be as precise [32, 33].

There are a few limitations and directions for further development of this work that needs to be discussed here. First, in our theoretical analysis we focused only on the MW and did not consider the effect of gas distribution in the model. Second, our analysis was not designed to predict the effects of different inspiratory flow patterns on the patient’s WOB during assisted mechanical ventilation. Although we calculated ventilator WOB, it remains a helpful parameter of the patient’s WOB later on during assisted ventilation when an effort is required for triggering and maintaining adequate inspiratory flows. The main contribution of our work is that to lower the ventilator WOB and consequently the risk of VILI, VCV with a square flow waveform is preferable than PCV.

Our conclusion can be used for patients with acute respiratory failure who totally depend on ventilators for breathing.

## Conclusion

Since in most intensive care patients, flow resistance is increased due to airway obstruction and/or tracheal intubation, there are numerous attempts to minimize WOB. The ventilator WOB or MW could be a helpful parameter of the patient’s WOB later on during assisted ventilation. Moreover, increasing MW is known as a risk factor for VILI. In this paper, we model the respiratory resistance and examine the effect of inspiratory flow patterns during VCV and PCV on MW. The resistive MW levels are compared between three flow profiles: square, and sinusoidal during VCV and decelerating during PCV.

Our study suggests the square profile for minimization of MW in mechanically ventilated patients either normal or COPD. Nonetheless, we find that by increasing I:E ratio in a constant tidal volume, resistive MW is decreased for all flow profiles. So, if a decelerating profile needs to be used, the MW can be reduced by increasing the I:E ratio.

Although our results do not suggest using the decelerating flow profile produced during PCV due to its high MW, its other beneficial aspects such as less pressure variability are not investigated in this study. To establish the optimum flow profile that can simultaneously minimize WOB, has the possible best gas distribution, and lowers the risk of VILI further research and model development is needed.

## Methods

Airway pressure during PCV can be described mathematically by Eq. 1 [22]. Airway pressure (P) increases exponentially to the pressure support level (PS) with a rising time (t_r_) and then remains constant until the termination of the inspiratory phase:

1 $$P = PS(1 - e^{{ - t/t_{r} }} ).$$

Using a simplified model for the respiratory system in which respiratory resistance and compliance are in series, inspiratory flow during PCV can be determined by Eq. (2). So, a decelerating flow waveform is generated which is related to the airway pressure, the airways resistance, and the respiratory time constant τ which is the product of airways resistance R and respiratory system compliance C:

2 $$f_{decelerating} = \frac{P}{R}e^{ - t/\tau } .$$

We derive the tidal volume *V*_T_ by integrating the flow equation in time as expressed in Eq. 3. Mathematica is used for the model calculations.

3 $$V_{T} = \int\limits_{0}^{{t_{i} }} {f_{{decelerating}} dt} = \int\limits_{0}^{{t_{i} }} {\frac{{PS(1 - e^{{ - t/t_{r} }} )}}{R}e^{{ - t/\tau }} dt = \frac{{PS}}{R}(\tau - \tau e^{{( - t_{i} /\tau )}} + (t_{r} \tau ( - 1 + e^{{ - (1/t_{r} + 1/\tau )^{{^{{t_{i} }} }} }} ))/(t_{r} + \tau )),}$$

4 $$f_{square} = \frac{{V_{T} }}{{t_{i} }},$$

5 $$f_{{{\text{sinusoidal}}}} = \frac{{V_{T} \pi }}{{2t_{i} }}\sin \,\left( {\frac{\pi t}{{t_{i} }}} \right).$$

Thus, the MW can be found by Eq. 6 and compared for different inspiratory flow waveforms:

6 $$MW = \int\limits_{0}^{{t_{i} }} {f.P\,dt} .$$

The closed-form expression for the MW corresponding to the decelerating waveform is given in Eq. 7:

7 $$\begin{aligned} MW_{decelerating} = & \int\limits_{0}^{{t_{i} }} {f_{decelerating} .P_{decelerating} dt} = \int\limits_{0}^{{t_{i} }} {\frac{{P^{2} }}{R}e^{ - t/\tau } dt} = \frac{{PS^{2} }}{R}\int\limits_{0}^{{t_{i} }} {(1 - e^{{ - t/t_{r} }} )\,^{2} e^{ - t/\tau } dt} \\ = \frac{{PS^{2} }}{R}\left[ \begin{gathered} (\tau e^{{t_{i} ( - 2/t_{r} - 1/\tau )}} ( - e^{{(2t_{i} /t_{r} )}} (t_{r}^{2} + 3t_{r} \tau - 2\tau^{2} (e^{{(t_{i} /\tau )}} - 1)) - t_{r} (t_{r} + \tau ) + 2t_{r} e^{{(t_{i} /t_{r} )}} (t_{r} + 2\tau ))) \hfill \\ /((t_{r} + \tau )(t_{r} + 2\tau )) \hfill \\ \end{gathered} \right]. \\ \end{aligned}$$

The airway pressure in VCV mode can be determined by the equation of motion which states that pressure required to deliver a volume of gas into the lungs is determined by the elastic and resistive properties of the respiratory system:

8 $$P^{VCV} = R.f + V/C.$$

In Eq. 8, f and V represent the air flow rate and the delivered tidal volume at the time of t. It should be noted that we have neglected inertance in this equation. Since we are interested in computing the resistive work, we could neglect inertance in our computations because it has been proved that when inertance is neglected, the resistance estimate contains no error [34]. The MW under VCV mode then can be determined by Eq. 9:

9 $$MW^{VCV} = \int\limits_{0}^{{t_{i} }} {f.(R.f + V/C} )dt = R\int\limits_{0}^{{t_{i} }} {f^{2} } dt + V_{T}^{2} /(2C).$$

The first term of the above equation is the resistive MW needed to overcome the resistance of the respiratory system and the second term is the elastic MW required to inflate the lung which is not dependent to flow waveform. Substituting Eqs. 4 and 5 into Eq. 9, we obtain the MW for square and sinusoidal flow waveforms shown in Eqs. 10 and 11, respectively:

10 $$MW_{square} = \int\limits_{0}^{{t_{i} }} {f_{square} (R.f_{square} + V_{T} /C} )dt = \frac{{RV_{T}^{2} }}{{t_{i} }} + V_{T}^{2} /(2C),$$

11 $$MW_{\sin usoidal} = \int\limits_{0}^{{t_{i} }} {f_{\sin usoidal} (R.f_{\sin usoidal} + V_{T} /C} )dt = \frac{{RV_{T}^{2} \pi^{2} }}{{8t_{i} }} + V_{T}^{2} /(2C).$$

Since the resistive MW is the only component of MW which is dependent on inspiratory flow waveform, it is enough to compare the resistive MW to investigate the effects of different waveforms on MW. Resistive MW is required to overcome the frictional resistance to air flow during mechanical ventilation that occurs due to the resistance of different compartments of the respiratory system, i.e., the endotracheal tube (ETT), lung airways, and pulmonary and chest wall tissue resistances.

In Eq. (9) it was assumed that the resistance is not dependent on flow characteristics. This assumption is valid only in laminar flow regime. However, airflows in mechanical ventilation increase to rates as high as 120 L/min. When the Reynolds number (Re) is below 2300, we expect the flow to be laminar, but for values larger than this threshold, the transition to critical or turbulent regime occurs. So, we have used Rohrer’s equation to describe the resistance of ETT, R_ETT,_ and also lung airways, R_L_ (Eq. 12):

12 $$R_{i} = K_{1,i} + K_{2,i} .f,$$

where *i* stands for ETT or L. *K*_1_ is related to the laminar resistance while K_2_ compensated for the turbulent effects on the resistance. The average values of K_1_, and K_2_ constants for ETT and lung airways both in normal and COPD subjects are listed in Table 3.

**Table 3:** Experimental Rohrer’s constants for ETT, and lung airways

	ETT [35]	Lung airways	
		Normal [36]	COPD [37]
K_1_ (cmH2O.L^−1^.s)	0.85	1.85	5.03
K_2_ (cmH2O.L^−2^.s^2^)	6.35	0.43	2.69

The resistance of the pulmonary and chest wall tissues, DR_L_ and DRw, can be expressed by the following exponential function [36]:

13 $$DR = R_{2} (1 - e^{{ - t_{i} /\tau_{2} }} ),$$

where *R*_2_ and *τ*_2_ are the resistance and time constant of the viscoelastic properties of the pulmonary or chest wall tissues. The mean values of *R*_2_ and *τ*_2_ for DR_L_ and DRw both in normal and COPD subjects are listed in Table 4.

**Table 4:** Experimental values of viscoelastic parameters in Eq. 13

Viscoelastic parameters	Pulmonary tissue		Chest wall tissue	
	Normal [36]	COPD [37]	Normal [36]	COPD [37]
*R*_2_ (cmH2O.L^−1^.s)	3.44	8.75	2.12	3.25
*τ*_2_ (s)	1.13	1.40	1.29	2.49

So, the total resistance of the respiratory system in mechanically ventilated patients can be found by Eq. 14:

14 $$R_{total} = R_{ETT} + R_{L} + DR_{L} + DR_{W} .$$

The total resistive MW can be computed by Eq. 15:

15 $$\begin{gathered} MW_{res,total} = \int\limits_{0}^{{t_{i} }} {R_{total} .f^{2} \,dt} \hfill \\ = \int\limits_{0}^{{t_{i} }} {R_{ETT} .f^{2} \,dt} + \int\limits_{0}^{{t_{i} }} {R_{L} .f^{2} \,dt} + (DR_{L} + DR_{w} ).\int\limits_{0}^{{t_{i} }} {f^{2} \,dt} . \hfill \\ \end{gathered}$$

We generate our results in the range of flow rates corresponding to three different tidal volumes of 0.5, 1, and 2 L. The reference condition here is defined by the rate of 15 respirations per minute and the I:E value of 1/4 which corresponds to an inspiration time of 1 s. The PS levels that deliver the desired tidal volumes of 0.5, 1, and 2 L during the PCV are 8.0, 16.1, and 32.1 cmH2O.

The time integrals expressed in Eq. 15, are computed for each of the flow relations expressed by Eqs. 2, 4, and 5 that represent decelerating, square, or sinusoidal flow waveforms, respectively.

**Fig. 3:**
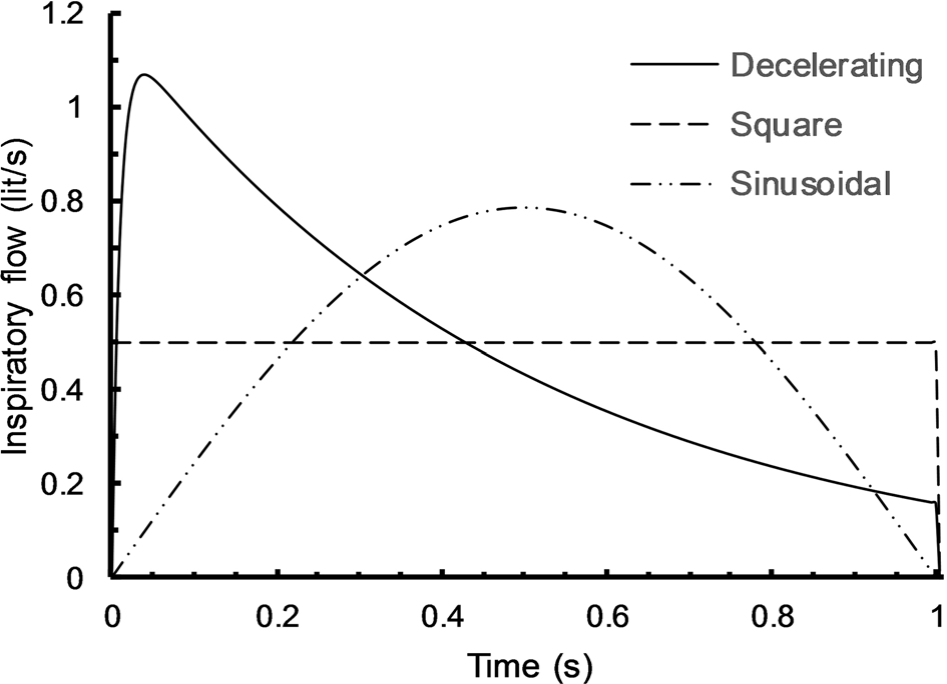
Three different flow waveforms under a tidal volume of 0.5 L, RR = 15/min, I:E = 1/4

Assuming a normal lung with *R* = 10 cmH2O/L/s, C = 0.1 L/cmH2O, and pressure rising time, *t*_r_ = 0.01 s, the three flow waveforms with a constant tidal volume of 0.5 L for reference values of PR and I:E parameters are shown in Fig. 3.

**Fig. 4:**
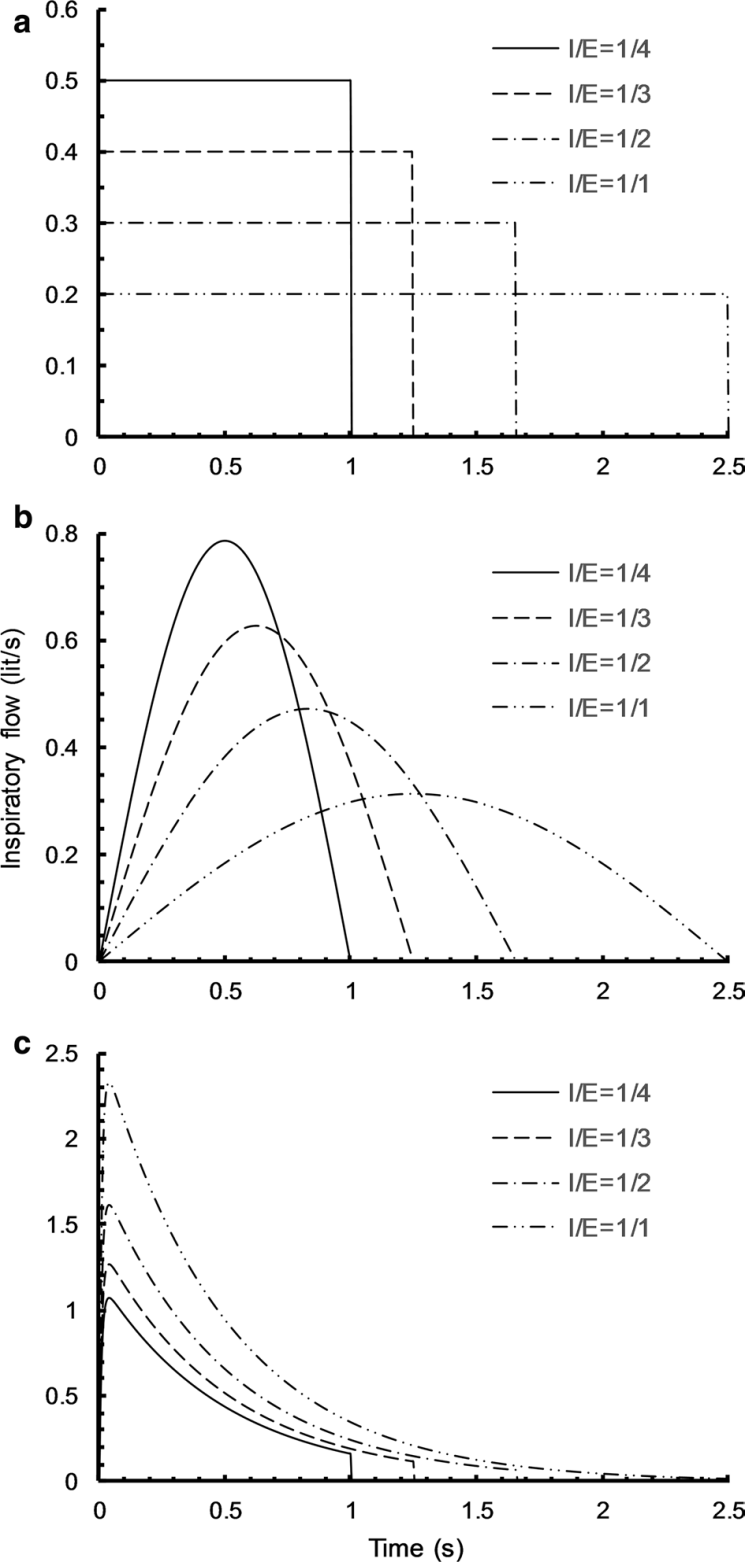
Effect of changing I:E ratio (1/4, 1/3, 1/2 and 1/1) on inspiratory flow profile under a constant tidal volume of 0.5 L, in **a** square flow waveform, **b** sinusoidal flow waveform, **c** decelerating flow waveform

Figure 4 illustrates how these profiles change as I:E ratio increases to 1 keeping the respiratory rate and tidal volume fixed at 15/min and 0.5 L, respectively.

## Data Availability

Not applicable.
